# A delayed-onset rectourethral fistula after intersphincteric resection for very low rectal cancer: a case report and literature review

**DOI:** 10.1186/s40792-015-0074-9

**Published:** 2015-08-29

**Authors:** Koji Komori, Kenya Kimura, Takashi Kinoshita, Seiji Ito, Tetsuya Abe, Yoshiki Senda, Kazunari Misawa, Yuichi Ito, Norihisa Uemura, Seiji Natsume, Jiro Kawakami, Yoshinori Iwata, Masayuki Tsutsuyama, Itaru Shigeyoshi, Tomoyuki Akazawa, Daisuke Hayashi, Yasuhiro Shimizu

**Affiliations:** Department of Gastroenterological Surgery, Aichi Cancer Center Hospital, 1-1, Kanokoden, Chikusa, Nagoya, Aichi 464-8681 Japan

**Keywords:** Rectourethral fistula, Rectal cancer, Intersphincteric resection

## Abstract

Rectourethral fistula is one of the complications that can occur after prostatectomy in the urologic discipline. However, a delayed-onset rectourethral fistula after intersphincteric resection (ISR) for low rectal cancer is extremely rare. Here, we report one such case in a 57-year-old man. After ISR for low rectal cancer with a diverting stoma (DS), the DS was closed. After approximately 1 year, frequent pneumaturia and right orchitis were observed. Results of contrast enemas and abdominal computed tomography examinations revealed a rectourethral fistula from an anastomosis to the urethra. The colonoscopic appearance revealed a pinhole fistula on the anastomotic line, with thick pus. We performed a transverse colostomy, and the pneumaturia and right orchitis were no longer observed. Two months later, colonoscopy, contrast enemas, and cystoscopy revealed no rectourethral fistula. To the best of our knowledge, our case is the first report of a delayed-onset rectourethral fistula after ISR.

## Background

Intersphincteric resection (ISR) is commonly performed for very low rectal cancer [1]. Previous reports showed that anastomotic leaks due to ISR occur in 3.4 % of cases [2]. Rectovaginal fistula occurs even less frequently after low anterior resection of rectal cancer [3]. On the other hand, rectourethral fistula frequently occurs after prostatectomy for prostate cancer, and its repair has been under intense investigation [4]. However, delayed-onset rectourethral fistula after ISR for low rectal cancer has not been reported previously. In the present report, we describe a very rare case of delayed-onset rectourethral fistula.

## Case presentation

**Fig. 1:**
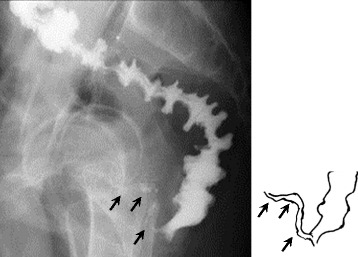
The left image shows contrast enemas revealing a rectourethral fistula (*black arrow*) from an anastomosis to the urethra. The right schema shows the rectourethral fistula (*black arrow*) from an anastomosis to the urethra

**Fig. 2:**
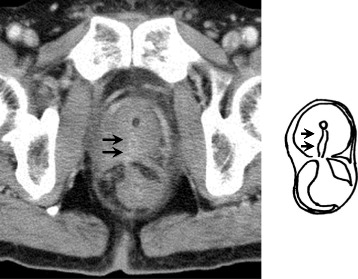
The left image shows a computed tomography scan revealing a rectourethral fistula (*black arrow*) from an anastomosis to the urethra. The right schema shows the rectourethral fistula (*black arrow*) from an anastomosis to the urethra

**Fig. 3:**
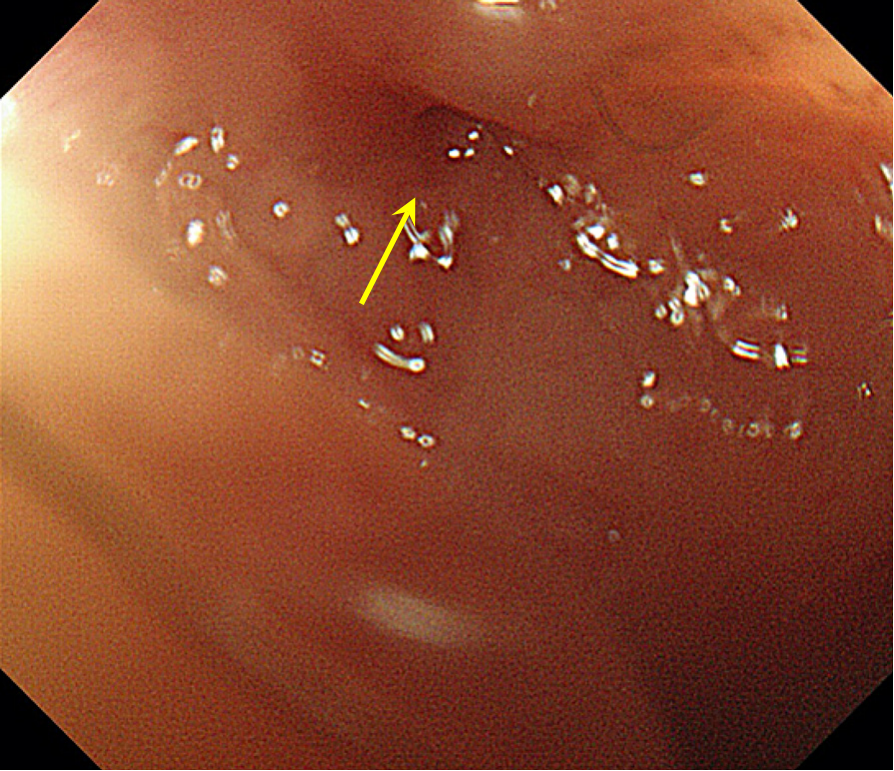
The colonoscopic appearance shows the pinhole fistula (yellow arrow) on the anastomotic line with thick pus

**Fig. 4:**
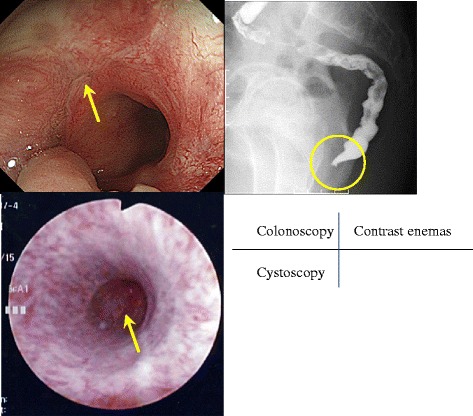
No rectourethral fistula was observed on colonoscopy (yellow arrow, *upper left image*), contrast enemas (yellow arrow, *upper right image*), or cystoscopy (yellow circle, *lower left image*)

The patient was a 57-year-old man who underwent ISR for low rectal cancer with a diverting stoma (DS) in May 2010 in our hospital (Aichi Cancer Center Hospital). Using 32 absorbable sutures, a manual end-to-end anastomosis was created. The primary tumor was located in the right lateral wall of rectum and 3 cm from the anal verge. He was discharged after an uneventful postoperative course. Histopathological examination revealed well-differentiated tubular adenocarcinoma with no lymph node metastasis, and it was classified as pStage II (pT3 N0 M0). Results of contrast enemas performed before the DS was closed revealed no anastomotic leak, so the DS was closed in September 2010. The patient had frequent defecation (about 10 times per day, Wexner’s constipation score, 0) without fecal incontinence for a period of time after this procedure. The frequent defecation was gradually declining (about twice per day, Wexner’s constipation score, 0). After approximately 1 year, frequent pneumaturia and right orchitis were observed. Results of contrast enemas revealed a rectourethral fistula from an anastomosis to the urethra (Fig. 1). Abdominal computed tomography (CT) examinations revealed a rectourethral fistula penetrating the prostate (Fig. 2). The colonoscopic appearance included a pinhole fistula on the anastomotic line, with thick pus (Fig. 3). We made a diagnosis of delayed-onset rectourethral fistula after ISR for very low rectal cancer. Transverse colostomy was performed, and in May 2011, the patient’s pneumaturia and right orchitis were no longer observed. Two months after the colonoscopy, contrast enemas and cystoscopy revealed no rectourethral fistula (Fig. 4). We encouraged the patient to have his transverse colostomy closed, but he preferred the transverse colostomy because the frequent defecation was intolerable. Although the period of time with frequent defecation was short, he could not accept the condition in the absence of the transverse colostomy. Approximately 5 years have passed since the initial operation, and the patient is completely without recurrence.

Our report has two points of great clinical significance. Firstly, the delayed-onset event occurred after ISR for very low rectal cancer is very rare. Secondly, our case is the first report about rectourethral fistula after ISR.

Anastomotic leaks have become troublesome in the surgical treatment of rectal cancer with or without a DS. Recently, in cases of low anterior resection for rectal cancer, Shiomi et al. reported that the frequency of anastomotic leak was 13.2 % in patients with DS versus 12.7 % in cases without DS; no significant differences were found [5]. On the other hand, in cases of ISR for rectal cancer, the frequency of anastomotic leak is relatively rare. Yamamoto et al. reported this frequency at 3.4 % [2], and Shiomi et al. reported it at 5.4 % [6]. In addition, Shiomi et al. reported that the anastomotic leak cases in the ISR were all early complications [6]. As noted below, we performed ISR cautiously to protect against the anastomotic leak. First, we confirmed the adequate blood flow in the surgical margin of colon. Secondly, the whole layer of colon and the sufficient amounts of external anal sphincter muscle were penetrated by 4–0 vicryl™ (absorbable suture), then the end-to-end anastomosis was accomplished tightly.

Symptoms associated with anastomotic leaks in previous reports include rectovaginal fistulas and seminal vesicle-rectal fistulas. Rectovaginal fistulas, the first type, have been frequently reported, and their repair has been adequately investigated and developed. Kosugi et al. reported that the gluteal-fold flap was a good technique for the repair of rectovaginal fistulas [3], but this technique has not yet proved practicable for the treatment of rectourethral fistulas because it is impossible to approach the side of the urethra. Seminal vesicle-rectal fistulas, the second type, have only been reported in very few cases. Kitazawa et al. reported 10 cases of seminal vesicle-rectal fistulas, and they defined rarely encountered complications [7]. In contrast, rectourethral fistula is one of the complications that occur after prostatectomy in the urologic discipline. There have been no previous reports describing a rectourethral fistula after ISR, as there is little available information in the gastroenterological surgery discipline.

The reports of delayed-onset anastomotic leaks are very few. August et al. reported three cases in association with bevacizumab therapy [8]. The interval between the original surgery and complications in those three cases ranged from 5 to 33 months. For our case, the interval between the original surgery and the complication was 15 months. Conversely, McDermott et al. reported that preoperative radiotherapy with or without concomitant chemotherapy was not a risk factor for anastomotic leaks [9]. Kosugi et al. hypothesized that colitis causes inflammation of the anastomotic site and perforation by frequent bowel movements, leading to the formation of seminal vesicle-rectal fistulas [3]. Similarly, we hypothesized that an unexplained micro-abscess formed at the anastomotic site, and a subsequent very small pinhole leak persisted (this leak was not observed on diagnostic imaging), and it was inflamed into the urethra through the prostate, completing the fistula.

The treatment options for rectal fistulas have been classified into two types: conservative measures and aggressive treatments. The former includes interventional radiology (percutaneous drainage) and colostomy, and the latter includes local repair [4] and re-anastomosis. In our case, a conservative measure, i.e., colostomy, was selected for treating the symptom of frequent defecation. However, if it fails to improve these symptoms, we will schedule local repair.

In our case, the patient rejected the closure of his colostomy. Therefore, it remains unclear whether this rectourethral fistula was healed; if the colostomy was closed, it would be clear that the rectourethral fistula made a resurgence.

## Conclusions

This report describes a very rare case of a delayed-onset rectourethral fistula after ISR as well as the advantages of conservative measures, i.e., colostomy, for the treatment of this complication. The pathogenesis was less pronounced; therefore, the operative manipulation of ISR must be performed cautiously.

## Consent

Written informed consent was obtained from the patient for the publication of this case report and any accompanying images. A copy of the written consent is available for review by the Editor-in-Chief of this journal.

## Abbreviations

CT: computed tomography
ISR: intersphincteric resection
DS: diverting stoma
